# Low Serum Albumin in Patients With Coexisting Cognitive Impairment Predicts Surgical Complications

**DOI:** 10.1093/geroni/igaa057.452

**Published:** 2020-12-16

**Authors:** Coco Victoria Tirambulo, Caitlyn McFadden, David Lieberman, Mindy Fain

**Affiliations:** 1 The University of Arizona Center on Aging, Tucson, Arizona, United States; 2 University of Arizona College of Medicine - Geriatrics, General Internal Medicine and Palliative Medicine, Tucson, Arizona, United States; 3 University of Arizona Center on Aging, Tucson, Arizona, United States

## Abstract

Cognitive impairment (CI, ~15-20%) and malnutrition (~38.7%) are common concerns among older adults ≥65 years. CI and malnutrition may be used as predictive risk factors for poor surgical outcomes. The 2012 ACS NSQIP/AGS Best Practice Guidelines for the preoperative assessment of geriatric surgical patients classify severe nutritional risk as either having a BMI < 18.5 kg/m2, serum albumin (SA) < 3.0 g/dL and/or unintentional weight loss > 10%-15% within 6 months. Using SA as a surrogate marker for malnutrition, we evaluated the relationship between CI, malnutrition, and risk for poor surgical outcomes in a geriatric population. Electronic medical record chart reviews of patients (≥65 years old) undergoing elective intermediate or high-risk surgery (IHRS), between 2016 and 2019 in Tucson, AZ, were conducted. Pre-and-post assessment factors such as cognitive status via mini-cog, laboratory markers (SA), and hospital complications were examined. Multivariate regression analyses were performed to determine the association between cognitive status, SA levels, and hospital complications. Of the 173 patients undergoing IHRS included in this assessment (mean age: 75.5±7.4 years, [60-93 years], 54.9% male), 42.8% experienced hospital complications. Multivariate regression analysis revealed cognitive impairment and low SA levels were significantly associated with this outcome (p<0.05), adjusted for age and gender. We demonstrated MCI and low SA levels are risk factors of postoperative hospital complications among older patients undergoing elective IHRS. Increased understanding of predictive factors can help enhance prevention efforts, aiding in improving patient experiences and reducing patient and hospital costs.

